# Biomechanical study of a newly developed continuous double knots technique compared with the 4-strand double-modified Kessler technique for flexor tendon repair

**DOI:** 10.1186/s40634-021-00404-4

**Published:** 2021-09-24

**Authors:** Sunton Wongsiri, Wongthawat Liawrungrueang

**Affiliations:** Department of Orthopedics, Faculty of Medicine, Prince of Songkla University, Songkhla, Thailand

**Keywords:** Biomechanical study, Flexor tendon injury, Flexor tendon repair

## Abstract

**Purpose:**

In this study we compare the biomechanical properties of a novel suture technique that we developed called the continuous double knots technique for repairing flexor tendon injuries with the standard 4-strand double-modified Kessler technique.

**Methods:**

This was an experimental study. Eighty porcine flexor digitorum profundus tendons were harvested and divided randomly into two groups of 40. The first group (*N* = 40) was repaired using the 4-strand double modified Kessler technique and the second group (*N* = 40) was repaired using our new continuous double knots technique. The two groups were randomly divided and the ultimate failure load (*n* = 20) and cyclic testing to failure (*n* = 20) were compared.

**Results:**

The mean ultimate failure load was 25.90 ± 7.11 (N) and cyclic testing to failure 88 ± 47.87 (cycles) for the 4-strand double modified Kessler technique and 34.56 ± 6.60 (N) and 189 ± 66.36 (cycles) for our new continuous double knots technique. The T-test revealed a significant difference between the 2 techniques (*p* < 0.05). In terms of biomechanical properties in tendon repair, the continuous double knots technique group had a higher tensile strength than the 4-strand double-modified Kessler technique group. There were also significant differences between the ultimate failure load and cyclic testing to failure for the flexor tendon sutures.

**Conclusions:**

The continuous double knots technique suture technique had significantly higher maximum tensile strength and cyclic testing than the 4-strand double modified Kessler technique in an in vitro study, and in thus an optional technique for flexor tendon repair.

## Introduction

The most common injury around the hand and finger is tendon injury [1], with the highest incidence in males aged between 20 and 29 years old [2]. Acute injury of the flexor tendon is more common than the extensor tendon and the standard treatment is primary end-to-end repair in the first 12–24 h. after the injury. However, one study reported that a delay in primary repair of 3 to 7 days after the injury increased the risk of complications [3]. Many studies have been conducted on biomechanical evaluation of different suture techniques to improve the tensile strength and cycle load to failure for early motion and the prevention of finger joint stiffness. The 4-strand core suture is the standard suture technique for tendon repair. The modified Kessler technique is commonly used for flexor tendon repair. Many new techniques have been suggested to overcome this weakness and improved the tensile strength of tendon repair [4–7]. A primary end to end flexor tendon repair technique must be strong enough for early passive mobilization rehabilitation. The field of flexor tendon repair still remains a challenge for hand surgeons who are always looking for the best compromise between suture strength and early mobilization. In this study we developed a novel suture technique we call the continuous double knots technique to repair flexor tendon injuries, and in this study compared the biomechanical properties, including maximum tensile strength and cyclic load to failure, with the 4-strand double-modified Kessler technique. We carried out an interesting experimental study comparing two 4-core suture techniques. The protocol is well explained and all the experimental steps conducted in the right path.

## Materials and methods

This study was approved by the Institutional Review Board of the Faculty of Medicine, Prince of Songkla University (IRB number EC 60–158–11-1). This was an experimental study in which tensile strength and cyclic load to failure were tested in vitro.

### Preparation and harvesting of animal tendons

80 tendons were harvested from 5-month old, 90–110 kg pigs. The tendons were taken from the deep flexor muscles of toes II and V (Tendo musculus flexor digitorum profundus ramsad digitorium II et V). The tendons were harvested by the Faculty of Natural Resources, Prince of Songkla University. A 7 cm length of tendon was harvested, beginning from the point the tendon was 5 mm in diameter. For the standardization of the tendons, a 5-mm hole was made in a plastic sheet, then the tendons were passed through the hole to identify the diameter. All harvested tendons were rechecked by re-inserting into the 5 mm^2^ diameter hole. All the tendons used in this study fit through the hole and had a smooth, continuous surface without any marks. The tendons were then stored in normal saline at 5 °C to maintain their natural properties and qualities. All harvested tendons were tested within 48 h. First the tendons were cut in half with a surgical blade to prepare for suturing. The tendons were randomly divided into two groups of 40, and then transected and repaired transected and repaired using the 2 techniques.

### Suturing techniques and suture material

**Fig. 1:**
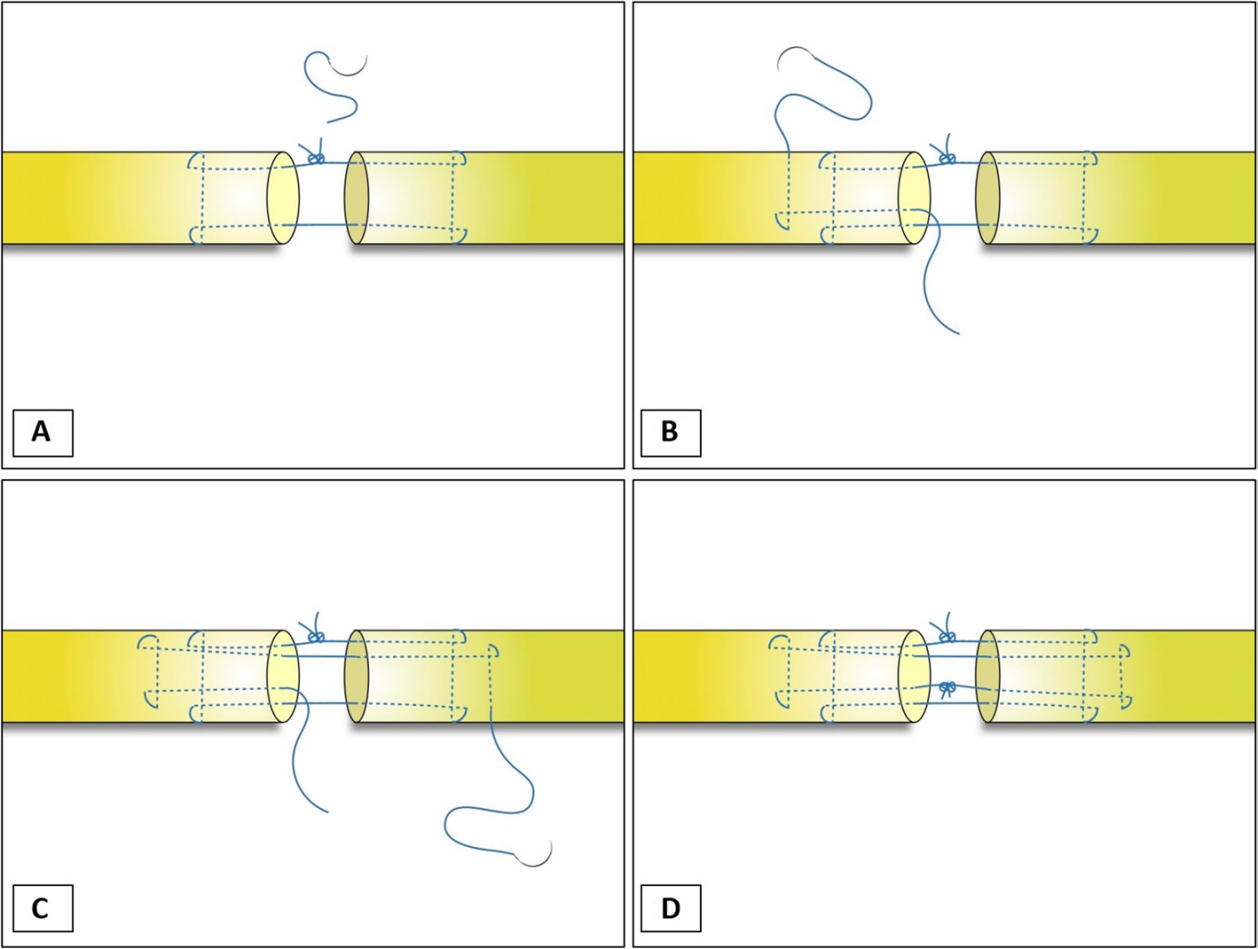
Showing the double-modified Kessler technique

**Fig. 2:**
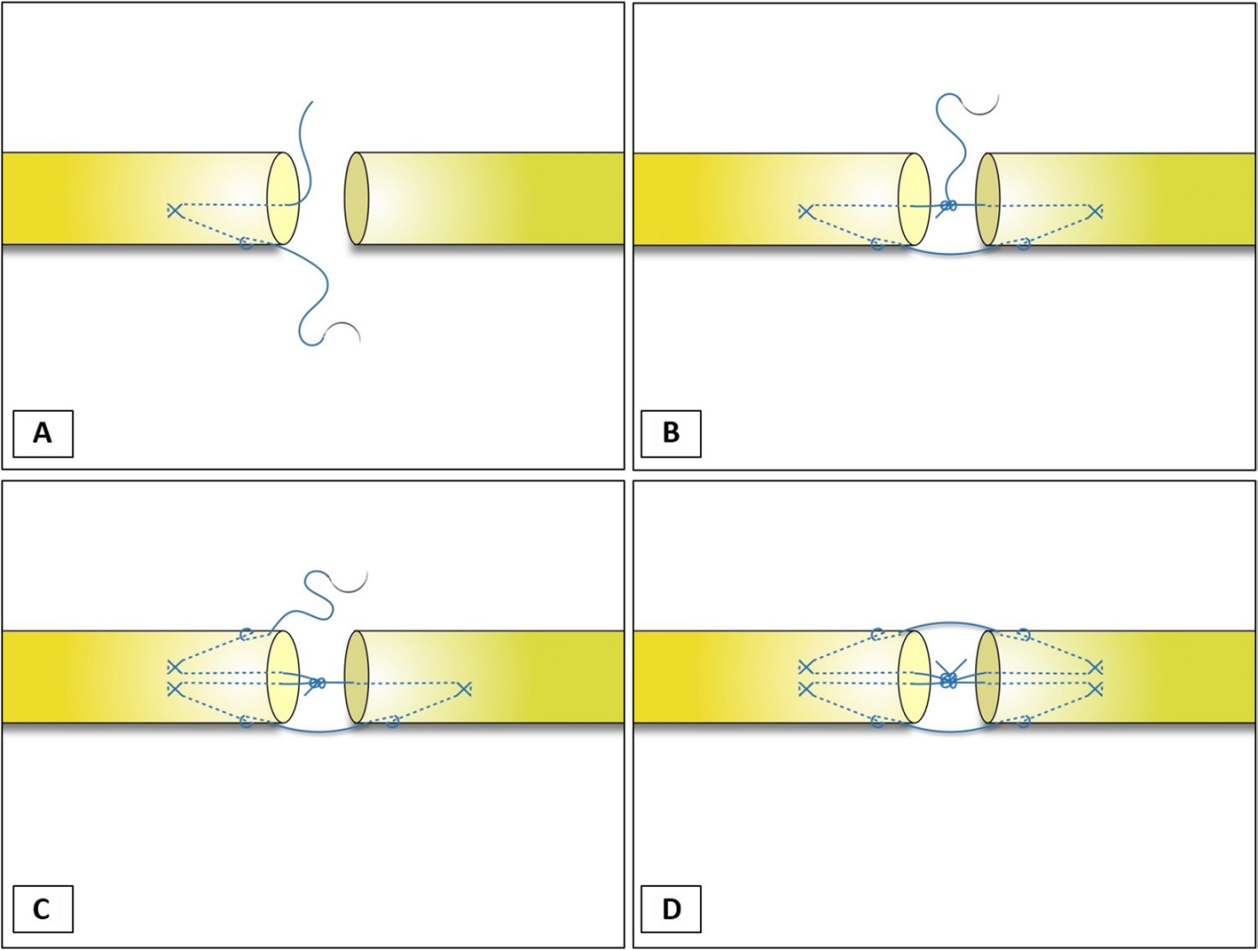
Showing the continuous double knots technique

**Fig. 3:**
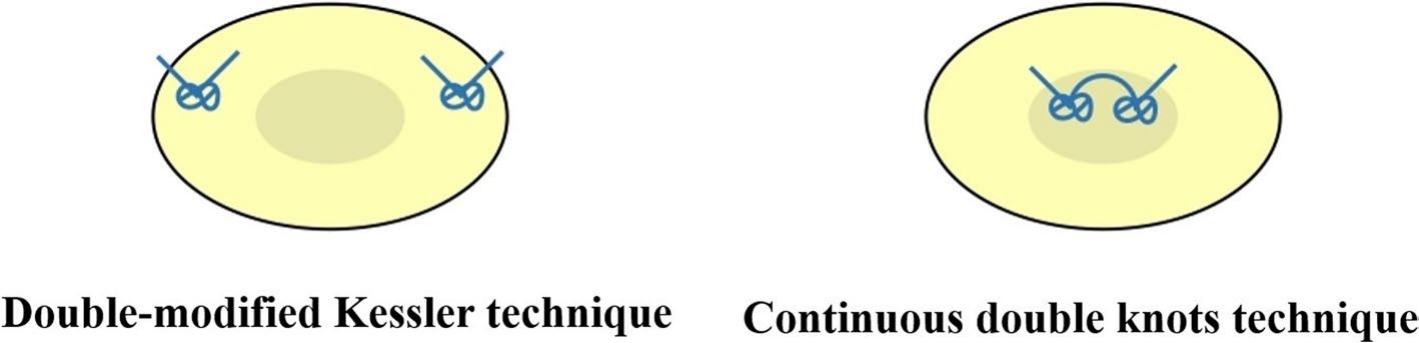
Showing the comparison of cross sectional of flexor tendon after repair by two 4-core suture techniques

The suture material used was polypropylene Size 4–0 (Ethicon®); only core sutures were used. In the 4-strand double-modified Kessler group, the tendon was repaired using a modified Kessler technique for the first loop. Then a knot was made and the suture cut. After the first loop was done, the second loop of the modified Kessler technique was made without the subsequent suture from the first loop. Then a knot was made and the suture cut, as shown in Fig. 1A-D. In the second group (Continuous double knots technique), the tendons were prepared in the same way as group 1. The tendon was repaired by cross-locking suture in the proximal and distal part of the tendons (Fig. 2A). The cross-locking of the distal part of both tendon ends can be a practical technique to control and optimize the length of the tendon to reduce the gap. The cross-locking of distal part of both tendon ends can control the tendon gap well by experienced hand surgeon. After the knot was tied (Fig. 2B), the repair continued in the second loop without cutting the suture (Fig. 2C). The final steps, suture knot of the second loop was tied to the first knot (Fig. 2D). A single hand surgeon did all the tendon repairs in both groups. The cross sectional of flexor tendon after repair in both groups were shown in Fig. 3.

### Biomechanical testing (ultimate strength and cyclic tensile strength)

**Fig. 4:**
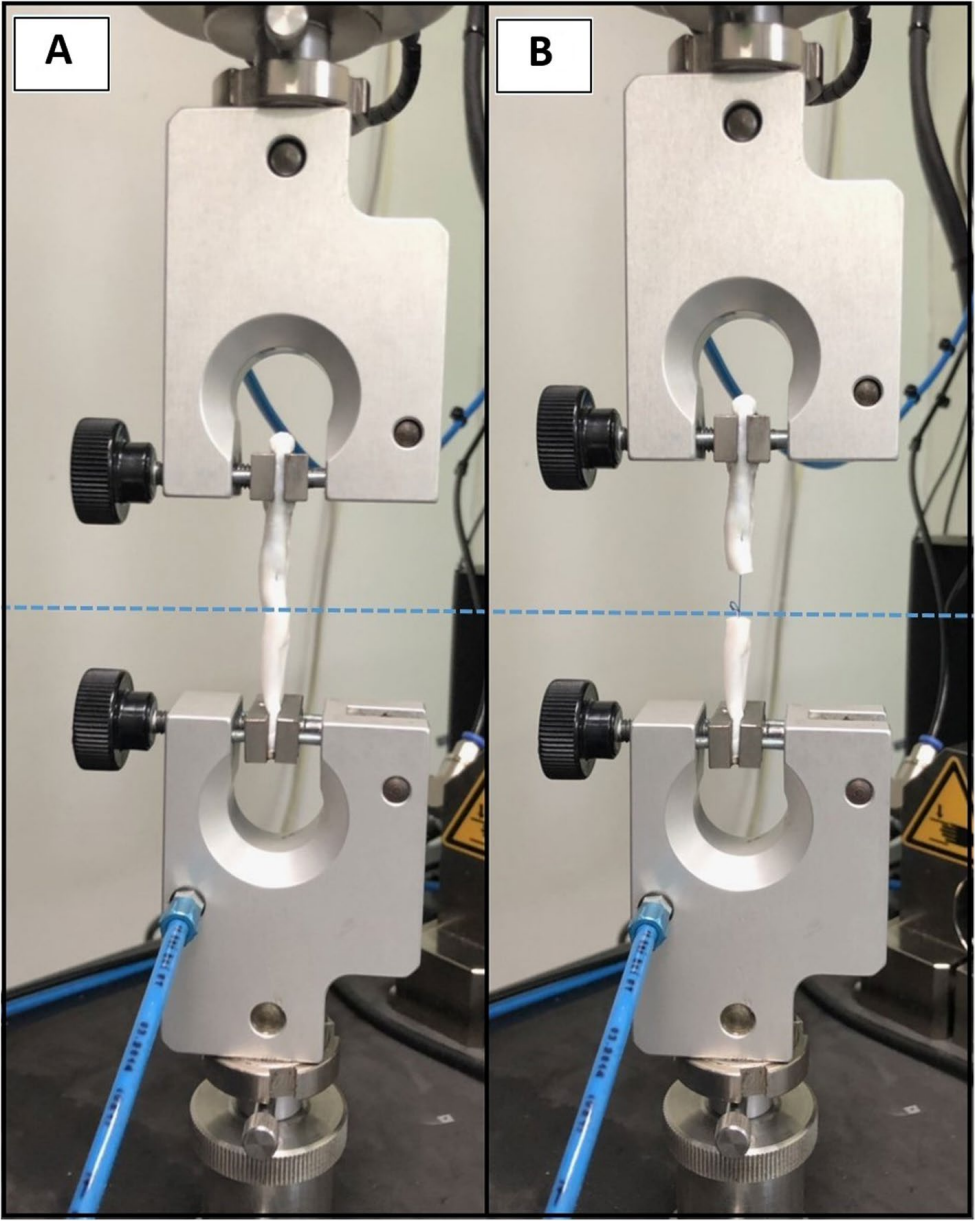
Universal Testing Machine (ZwickRoell Z010), 2 grippers were maintained (3A), and the tensile strength was recorded until the suture thread was broken (3B)

The sutured tendons were kept at 5 °C and hydrated until the strength and cyclic testing. The sutured tendons were examined using a Universal Testing Machine (ZwickRoell Z010) at the Office of Scientific Instruments and Testing. Ultimate tensile strength testing used the standard protocol in accordance with previously published papers [8, 9]: the vertical distance between the 2 grippers (Fig. 4A) was maintained at 25 mm. The 2-N preload was applied to condition the repaired tendons for 5 cycles, which were then pulled until they failed. The strain rate of the mechanical tester was set at 20 mm/min [8, 9]. The maximum strength the tendon could tolerate until the suture thread broke was recorded (Fig. 4B). A high-resolution camera was used to record the failure and the corresponding force was measured.

Fatigue strength was the sum of the product of the number of cycles and the applied load that was used the cyclic testing protocols for flexor tendon repairs, we used the standard protocol from Chang et al. [10], who proposed a cyclic tensile strength test using 2-N for preload then a cyclic load of 15 N to simulate passive mobilization and 2000 cycles at a frequency of 0.2 Hz. until the suture thread broke. This cyclic tensile strength testing was in accordance with previously published papers.

### Statistical analysis

All data were collected and analyses conducted using the R Program version 3.1.0 (R Foundation for Statistical Computing, Vienna, Austria). The normally distributed data were analyzed with the Kolmogorov–Smirnov test. Data of ultimate strength and number of cycles to failure in the double-modified Kessler technique group compared with the continuous double knots technique group were normally distributed and comparisons of means and variances were carried out using Student’s T-test with a cut off of *P*-value less than 0.05 for significance.

## Results

The results of ultimate tensile strength and number of cycles to failure showed normal distribution. The 2-Sample Student’s t-test was used to compare means and a significant difference was found in ultimate tensile strength and cyclic testing to failure.

### Ultimate tensile strength

The ultimate tensile strength are shown in Table 1. The mean of the ultimate tensile strength in the double modified Kessler technique (group1) and the continuous double knots technique (group 2) were 25.90 ± 7.11 (N) and 34.56 ± 6.60 (N), respectively, which was significant (*p* < 0.001).

**Table 1:** The mechanical properties of the double-modified Kessler technique compared with the continuous double knots technique

Type of tensile strength	Double modified Kessler technique (Group 1)	Continuous double knots technique (Group 2)	*P*-value(p)
Ultimate strength (N)	25.90 ± 7.11 (*n* = 20)	34.56 ± 6.60 (*n* = 20)	*p* ≤ 0.001*
Cyclic testing to failure (cycles)	88 ± 47.87 (*n* = 20)	189 ± 66.36 (*n* = 20)	*p* ≤ 0.001*

*P-value less than 0.05 for significance (95% confidence intervals in parentheses)

### Cyclic testing to failure

The cyclic testing to failure results are shown in Table 1. The mean of cyclic testing to failure with the double modified Kessler technique (group1) was 88 ± 47.87 (cycles) and with the continuous double knots technique (group 2) was 189 ± 66.36 (cycles).The difference was again significant (*p* < 0.001).

### Mechanism of failure

**Fig. 5:**
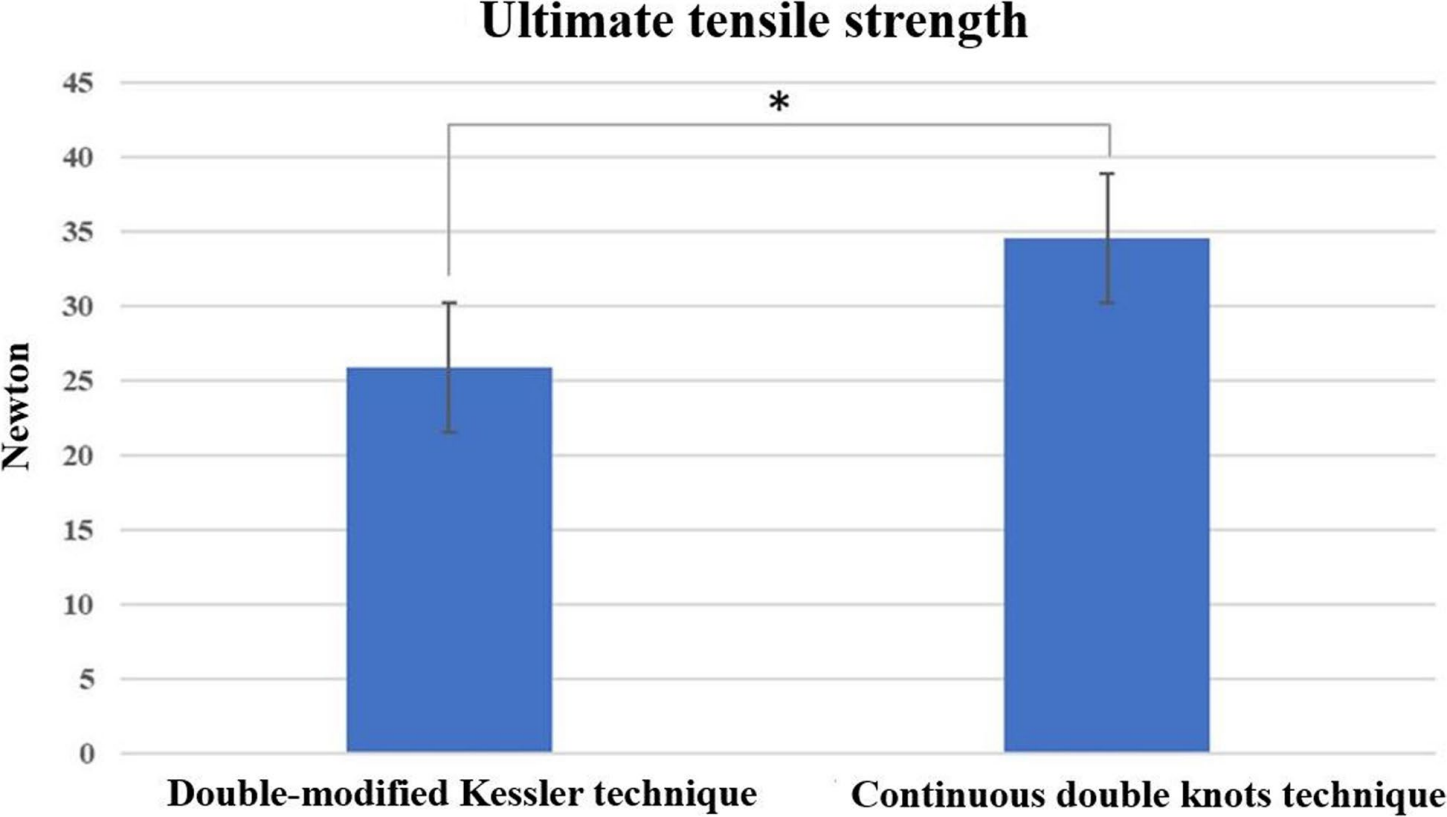
Comparison of ultimate tensile strength shown for each tendon repair technique. The difference between the double-modified Kessler technique and the continuous double knots technique was statistically significant

**Fig. 6:**
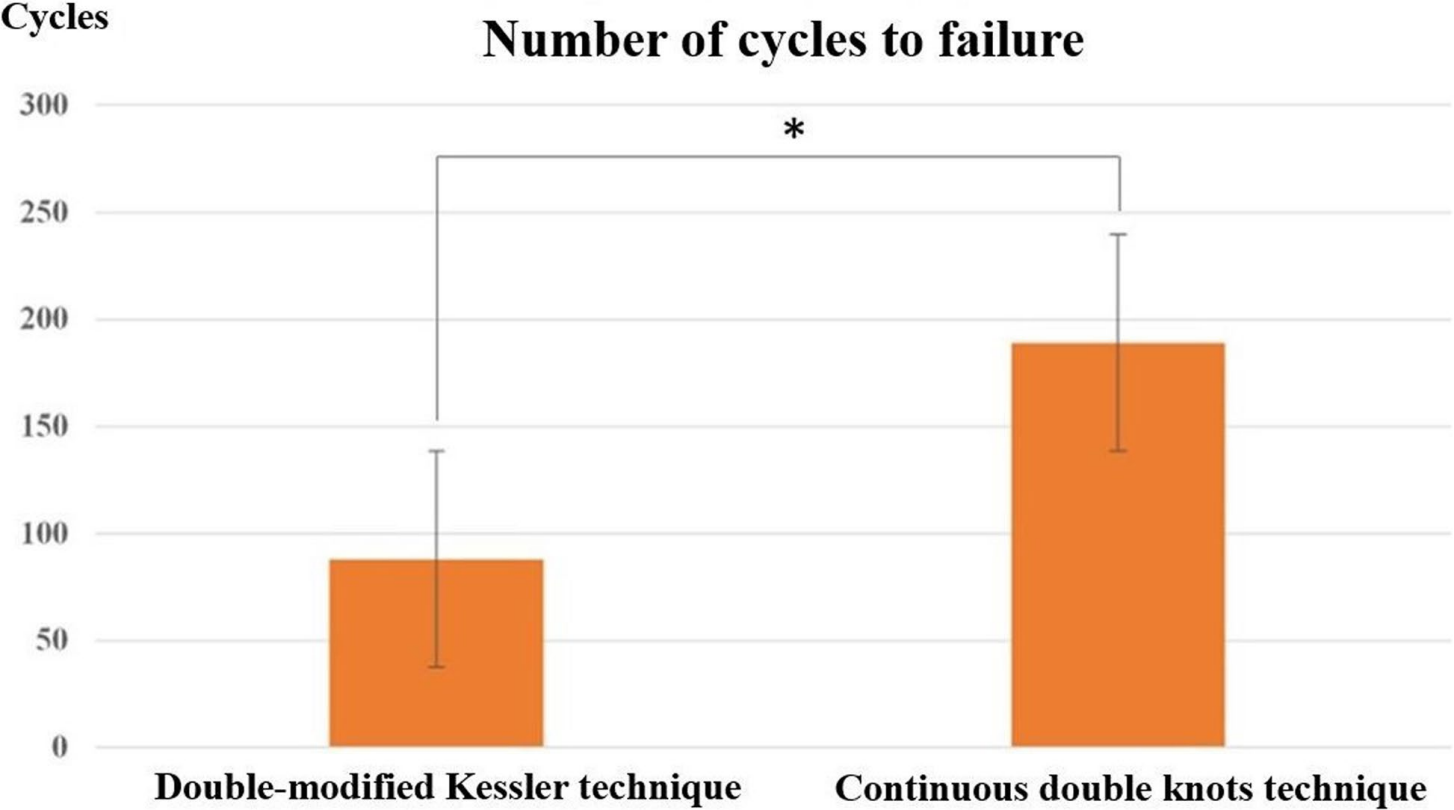
Comparison of number of cycles to failure shown for each tendon repair technique. The difference between the double-modified Kessler technique and the continuous double knots technique was statistically significant

The failure modes of the double-modified Kessler technique and the continuous double knots technique are shown in Table 2. The T-test revealed 2 significant differences in the 2 techniques (*p* < 0.05). The biomechanical analysis showed that tendon repairs in the continuous double knots technique group had higher tensile strength than in the 4-strand double-modified Kessler technique group. There were also significant differences between the groups in the ultimate failure load (Fig. 5) and cyclic testing (Fig. 6) to failure for flexor tendon sutures.

**Table 2:** Failure modes of the double-modified Kessler technique compared with the continuous double knots technique

Type	Double suture breakage	Double suture pullout	Double knot failure	One suture breakage + one suture pullout	One suture breakage + one knot failure	One suture pullout + one knot failure
**Group 1: Double modified Kessler technique with Ultimate strength test (*n*** **= 20)**	10	1	1	6	1	1
**Group 1: Double modified Kessler technique with Cyclic test (*n*** **= 20)**	9	2	1	5	2	1
**Group 2: Continuous double knots technique with Ultimate strength test (*n*** **= 20)**	8	4	0	7	0	1
**Group 2: Continuous double knots technique with Cyclic test (*n*** **= 20)**	7	5	0	8	0	0

The modes of failure were double suture breakage, double suture pullout, double knot failure, one suture breakage + one suture pullout, one suture breakage + one knot failure, and one suture pullout + one knot failure. The most common failure mode in both groups was double suture breakage.

## Discussion

Penetrating hand trauma is common, and in this kind of trauma the tendons of the hand or wrist are frequently injured. The flexor tendon repair was the standard procedure that it is very common for general orthopedic or hand surgeon but the outcomes are optimal due to suture technique and surgeon experience. Recently, many studies have tried new techniques in order to improve the tensile strength. In this study, we focused specifically on the technique used in the 4-strand suture method, and our findings can be combined with other studies on other techniques to determine the most effective surgical technique for tendon repair.

The initial strength of a repaired tendon depends on the number of suture strands crossing the repair site, core suture purchase length, anchoring technique, lock diameter and core suture material [5, 9]. The suturing method is also important in terms of post-operative outcomes such as restriction in motion. The purpose of this study was to assess a novel surgical suturing technique for flexor tendon repair compared with the 4-strand double-modified Kessler technique that is commonly used in orthopedics. We applied a single continuous suture to repair the tendon, adding surgical knots in the same place as the surgical knot at the repair sites(Fig. 2) theorizing that this technique would improve both max failure load and cyclic testing to failure. The testing protocol we chose was based on the 4 strands that comprise the core components of the tendon repair because the 4-core strand suture is the standard. The repair site gap formation is a very important issue in end-to-end flexor tendon repairs, as studies have shown that it can be associated with poor clinical outcomes [9]. A previous study found that increasing the number of core sutures used in the standard technique improved the ultimate strength of the core strand sutures [6, 8, 11]. The reason we chose to examine biomechanical tendon repair was that we believe this technique has not achieved its full potential in terms of technique development. Therefore, we applied a continuous suture to repair the tendon, adding surgical knots in the same place as the surgical knot at the repair sites in order to increase the strength in the repair site to improve tension resistance in the early motion using a simple suture technique. The biomechanical properties examination showed that tendon repair in the continuous double knots technique group had a higher tensile strength than in the 4-strand double-modified Kessler technique group. There were significant differences between the groups in terms of ultimate failure load and cyclic testing to failure for the flexor tendon sutures. The most common failure mode of tendon repair was suture breakage. Interestingly, the knot failure was less likely occur in the continuous double knots technique group compared with the double-modified Kessler technique group. This implies that the continuous double knots technique results in more secure knots. In this study, we found that knotting together of the continuous double knots suture technique can improve strength more than tie knots separately. The first possible reason for this is that, the two sutures could help share the load and balance the tension. The rate of double suture breakage when using the continuous double knots suture technique in our study is lower than in the double modified Kessler technique. Usually, two separate sutures will have different tensions, so the higher tension suture tends to rupture first. When knotted together, the higher tension suture can pass tension to the second suture and reduce the tension on itself.

The continuous double knots technique is an addition to the modified Kessler technique. This technique improved the strength of the procedure. Hence, the improved strength resulting from the knotting together of two separate sutures could be helpful for other techniques where two separate stitches could also be strengthened by tying the knots together. Other tendon repair techniques such as the Strickland technique or Becker technique might also benefit from this type of double knotting technique.

For the second reason, we observed that the broken sutures were not always completely separated from the tendon. One side of the broken suture might still be attached to the knot while the other side remained attached, but with reduced tension, as shown in Table 2. This indicates that the ruptured suture still carries a partial load but cause the other side to accept increased tension. In this situation, even though the tension is only slightly reduced, it can still slow the failure of the remaining suture. However, it may be reduced tension only partially but it is useful and may slows down the failure of last remained suture. Finally, we noted that the when the knots remained intact, they were difficult to pull out. Failure mode of knot failure in the continuous double knots technique was lower than double modified Kessler technique. In our technique found that tying the knots together would save time and reduce steps. Therefore, this technique may be more useful for other suture materials and other repair methods knotting that are slippery and tend to be easily pulled out. As same as the 4-strand cross-locked cruciate flexor tendon repair technique (Adelaide technique) has been shown to have comparably high resistance to gap formation and ultimate tensile strength [12, 13].

However, Adelaide repair technique had the single knot in two loops and cross-locking in proximal only [13]. This continuous double knots technique tie each knot of the two loops together and lock the proximal and distal part of the tendons. The cross-locking of the distal part of both tendon ends can be a practical technique to control and optimize the length of the tendon to reduce the gap formation.

Primary flexor tendon repair is an important surgical skill in orthopedic surgery [14]. The technique for end-to-end repair of flexor tendons should achieve a strong enough repair for early mobilization and healing in order to prevent adhesions. The evidence from other studies indicated that the strength of a repair is almost directly proportional to the number of knots and core sutures [15–17]. A previous in vivo study found that the effect of suture knot location on tensile strength after flexor tendon repair had no deleterious effects on tensile strength and may even have stimulated tendon healing [18]. However, the formation of a gap at the tendon repair site represents a dehiscence of the repair. Other studies have suggested that the formation of such a gap could lead to flexor tendon adhesions, decreased tendon glide, and consequent digital stiffness [19–23]. Gelberman et al. [24] found that repaired flexor tendons with a gap of less than 3 mm had increased strength compared to those with a gap of greater than 3 mm. Current evidence shows good to excellent outcomes in 75% of primary flexor tendon repairs [21, 25]. The rupture rate is 4–10% of primary flexor repairs [21, 25]. Active rehabilitation protocols give a better range of movement, smaller flexion contractures, and greater patient satisfaction when compared to the passive rehabilitation protocols [20, 21, 26].

The limitation of this study was that it was only a biomechanical study of core sutures. After tendon repair, the conditions of the paratenon and excursion are as important as the gap. This technique needs to be validated in a clinical trial in the future study.

## Conclusion

An in vitro study found that the newly developed continuous double knots technique is an optional technique for flexor tendon repair. The maximum tensile strength and cyclic testing were significantly higher in this group. The authors would like to call this newly technique as Wongsiri-Wongthawat flexor tendon repair technique.

## Data Availability

The datasets used and/or analysed during the current study are available from the corresponding author on reasonable request.
